# Analysis of Risk Factors for Adjacent Segment Degeneration after Minimally Invasive Transforaminal Interbody Fusion at Lumbosacral Spine

**DOI:** 10.1155/2022/4745534

**Published:** 2022-04-21

**Authors:** Shengtao Dong, Jie Li, Xiaomeng Jia, Jieyang Zhu, Yaoning Chen, Bo Yuan

**Affiliations:** ^1^Department of Spine Surgery, the Second Affiliated Hospital of Dalian Medical University, Dalian 116023, China; ^2^Department of Gastrointestinal Surgery, the Second Affiliated Hospital of Dalian Medical University, Dalian 116023, China; ^3^Department of Oncology, the Second Affiliated Hospital of Dalian Medical University, Dalian 116023, China; ^4^Department of Reparative and Reconstructive Surgery, the Second Affiliated Hospital of Dalian Medical University, Dalian 116023, China

## Abstract

**Background:**

Adjacent segment degeneration (ASD) has been considered as a serious complication from changes in the biological stress pattern after spinal fusion. The sagittal balance significantly associated with lumbar loading is largely dependent on L5-S1 segment. However, the evidence indicating risk factors for radiological and symptomatic ASD after minimally invasive transforaminal interbody fusion (MIS-TLIF) remains insufficient.

**Methods:**

This single-central retrospective study recruited patients with lumbosacral degeneration receiving MIS-TLIF at the L5-S1 level from January 2015 to December 2018. The targeted variables included demographic information, radiological indicators, surgery-related parameters, and patient-reported outcomes (PROs) extracted from the electronic medical system by natural language processing. In these patients, a minimum of 3-year follow-up was done. After reviewing the preoperative and postoperative follow-up digital radiographs, patients were assigned to radiological ASD group (disc height narrowing ≥3 mm, progressive slipping ≥3 mm, angular motion >10°, and osteophyte formation >3 mm), symptomatic ASD group, and control group. We identified potential predictors for radiological and symptomatic ASD with the service of stepwise logistic regression analysis.

**Results:**

Among the 157 consecutive patients treated with MIS-TLIF in our department, 16 cases (10.2%) were diagnosed with radiological ASD at 3-year follow-up. The clinical evaluation did not reveal suspicious risk factors, but several significant differences were confirmed in radiological indicators. Multivariate logistic regression analysis showed postoperative PI, postoperative DA, and ∆PI-LL in radiological ASD group were significantly different from those in control group. Nevertheless, for patients diagnosed with simultaneously radiological and symptomatic ASD, postoperative DA and postoperative PT as risk factors significantly affected the clinical outcome following MIS-TLIF.

**Conclusion:**

In this study, while approximately 10% of lumbosacral degenerations develop radiographic ASD, prognosis-related symptomatic ASD was shown not to be a frequent postoperative complication. Postoperative PI, postoperative DA, and mismatched PI-LL are risk factors for radiological ASD, and postoperative DA and postoperative PT are responsible for the occurrence of symptomatic ASD following MIS-TLIF. These radiological risk factors demonstrate that restoration of normal sagittal balance is an effective measure to optimize treatment strategies for secondary ASD prevention.

## 1. Introduction

Spinal fusion has become the mainstay of treatment for degenerative spinal disorders owing to significantly improving vertebral balance and promoting intervertebral fusion [1, 2]. Minimally invasive TLIF (MIS-TLIF), first introduced by Foley et al. in 2002, kicked off the minimally invasive era of spinal surgery [3]. Since then, minimally invasive techniques have become popular as a result of decreased intraoperative blood loss, reduced length of stay, and rapid postoperative mobilization [4–6]. In parallel, intervertebral fusion is thought to increase the biological stress on adjacent unfused segments and the consequent risk of degeneration by reconstructing the vertebrae-disc space [7–9]. Based on functional outcomes, adjacent disc degeneration has been classified as radiographic ASD and symptomatic ASD, with incidences of 1–20% and 10–80%, respectively [10].

Meanwhile, the L5-S1 segment, the transitional region of the lumbosacral spine, is responsible for forty percent of the overall lumbar lordosis, consisting of the lordosis angle of the L5 vertebra and the disc angle of L5-S1 [11]. Considering that biological stress is the main trigger for the incidence of ASD, it is necessary to assess the long-term complications in patients with spinal degeneration undergoing MIS-TLIF in the lumbosacral spine. The aim of this study was to analyze the risk factors associated with radiological and symptomatic ASD in patients following classical MIS-TLIF.

## 2. Materials and Methods

### 2.1. Patient Population

We retrospectively analyzed clinical data from consecutive patients with lumbosacral degeneration. All cases were treated with MIS-TLIF at L5-S1 segment in our institution from January 2015 to December 2018.

The inclusion criteria for recruited patients are shown below: 1. the diagnosis of L5-S1 single-level degenerative spinal diseases (including disc herniation, spinal stenosis, and spondylolisthesis); 2. the operation of MIS-TLIF and percutaneous screw fixation without any other segmental interbody fusion or approach; 3. the minimum follow-up of 3 years; 4. comparable preoperative, postoperative, and follow-up radiological data available. Patients with incomplete data (*N* = 13), or preexisting adjacent segment degeneration (*N* = 41), or severe spondylolisthesis (grade 3 or higher) and scoliosis (degree>10) (*N* = 8), or spinal tumor, fracture, infection (*N* = 26), or follow-up lost (*N* = 10) were excluded.

Demographic information and surgery-related parameters were obtained from the electronic medical system (EMS). Standard preoperative radiological examinations are mandatory to optimize the surgical strategy, including standing lumbar anteroposterior (AP) and lateral and/or flexion and/or extension radiography, computed tomography (CT), and 1.5 T and/or 3.0 T magnetic resonance imaging (MRI), and radiological data were measured through picture-archiving system. The follow-ups were performed through the outpatient system and telephone surveys. This study was reviewed and approved by the ethics committee of our hospital.

### 2.2. ASD and Parameters Measurement

Patients' demographic information with respect to age, sex, body mass index (BMI), osteoporosis status, smoking status, diabetes, and diagnosis were recorded. Surgery-related parameters classified patients according to whether they underwent laminectomy and cement augmentation. Patient-reported outcomes (PROs) consisting of VAS and ODI scores reflected the status of clinical symptomatic remission.

**Figure 1:**
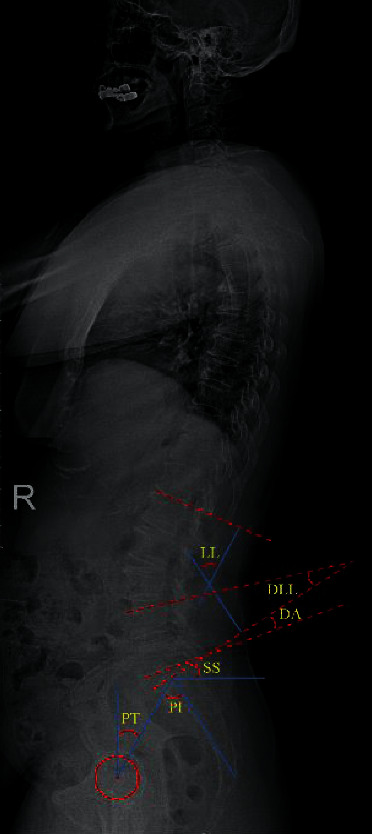
Schematic diagram of spinopelvic and lumbar indicators measurement. *PI*: angle between sacral plate and vertical line, *PT*: angle between femoral head-sacral plate midpoint line and longitudinal axis, *SS*: angle between sacral plate and the horizontal axis. *LL*: angle between L1 and sacral plate, *DLL*: L4 superior endplate to S1, *DA*: L5 lower endplate to S1, and *∆PI-LL* is calculated as the difference between PI minus LL.

Dynamic radiological evaluation was performed preoperatively, 2 days postoperatively, and at the final follow-up in the free-standing position X-ray. The measurements (Figure 1) involved spinopelvic indicators: pelvic incidence (PI), pelvic tilt (PT), and sacral slope (SS), and lumbar indicators: lumbar lordosis (LL), distal lumbar lordosis (DLL), disc angle (DA), and the lumbopelvic match (PI-LL). Meanwhile, the radiological complication of instrumental MIS-TILIF, namely, cage subsidence, was also recorded.

Patients were followed for a minimum of 3 years and, after radiological and clinical evaluation, were assigned to the radiological ASD group (disc height narrowing ≥3 mm, progressive slipping ≥3 mm, angular motion >10°, and osteophyte formation >3 mm) and/or the symptomatic ASD group.

### 2.3. Surgical Technique

**Figure 2:**
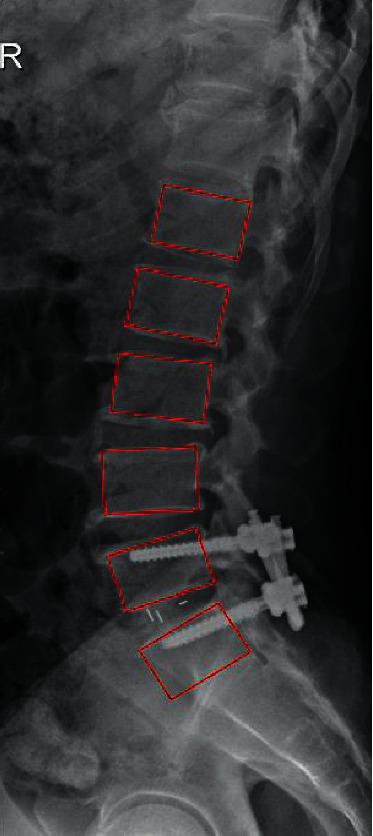
Schematic of a 63-year-old female patient following instrumented MIS-TLIF.

All single-segment MIS-TLIFs at L5-S1 were performed by the same senior spine surgeon. After induction of general anesthesia, the patient was placed prone on a radiolucent operating table with cushions under the pelvis and chest to ensure a posture reduction position. After the surgical approach was mapped out with the assistance of the C-arm, a minimally invasive 4 cm posterior incision was made. Subsequent visualization was accomplished by a series of continuous dilators to expand the fascia and separate the underlying paraspinal muscles. Based on clinical symptoms, the side with significant stenosis was decompressed transversely and an intervertebral cage filled with allograft bone was placed into the debrided interbody space to obtain a maximal disc height recovery. Under fluoroscopic guidance, percutaneous screws are inserted and compression of the rods is carried out to obtain the most feasible localized lordosis (Figure 2). Cement injection depended on the surgeon's evaluation of the preoperative findings and intraoperative bone quality.

### 2.4. Statistical Analysis

Differences between groups were analyzed by Fisher exact test, independent *t*-test, Mann-Whitney *U* test, Chi-square test, and Wilcoxon signed ranking test. Continuous and categorical variables were shown as mean ± standard deviation (SD) and relative frequencies and percentages, respectively. Meanwhile, stepwise logistic regression analysis was performed to analyze the susceptible risk factors for radiological and symptomatic ASD. All statistics were carried out using SPSS version 22.0 (IBM SPSS, Armonk, New York) and statistically significant differences were defined as *p* < 0.05.

## 3. Results

Among the 157 consecutive patients treated with MIS-TLIF in our department, 16 cases (10.2%) were diagnosed with radiological ASD at 3-year follow-up. Within the radiological ASD group, 6 cases (3.8%) reported unfavorable clinical symptoms and were assigned to the symptomatic ASD group. The comparison of demographic information and surgery-related parameters, as shown in Table 1, failed to find any statistical differences (*p* > 0.05). All preoperative and postoperative follow-up radiographic data were measured by two experienced spine surgeons and were summarized in Table 2. According to previous reports, a threshold of 10° was used to classify patients with a ∆PI-LL greater than 10° as a mismatch group and the remaining patients as a match group. The postoperative PI and postoperative PT were significantly higher and postoperative DA, postoperative LL, and frequency of matched group were significant lower within ASD patients. Furthermore, a significant improvement in patient-reported outcomes was observed over a follow-up period lasting three years, with even patients presenting with adjacent vertebral degeneration conveying to us a relatively better symptom relief and lower clinical scores. This series of evidence may support that the surgical technique of MIS-TLIF with percutaneous screw fixation at L5-S1 level provides acceptable long-term outcomes for patients (Table 3).

With comparison of selected indicators between radiological ASD and control patients, multivariate logistic regression analysis showed statistically significant differences in postoperative PI (OR 0.842, 95% CI 0.742–0.956, *p* = 0.008), postoperative DA (OR 0.804, 95% CI 0.703–0.919, *p* = 0.001), and ∆PI-LL (mismatched group, OR 4.370, 95% CI 1.015–18.816, *p* = 0.048) as in Table 4. Nevertheless, for patients diagnosed with simultaneously radiological and symptomatic ASD, postoperative DA (OR 0.777, 95% CI 0.634–0.951, *p* = 0.015) and postoperative PT (OR 1.246, 95% CI 1.009–1.539, *p* = 0.041) as risk factors significantly affected the clinical outcome following MIS-TLIF as in Table 5.

## 4. Discussion

Compared to conventional open posterior techniques, MIS-TLIF is a well-established procedure for the treatment of lumbar degeneration for decreased intraoperative blood loss, reduced length of stay, rapid postoperative mobilization, and early reengagement [4–6]. However, the debate on long-term postoperative complications has been consistent [9]. Previous studies have reported a distinct association between the development of ASD and postoperative vertebral mechanical alterations [10]. In the last decade, restoring the sagittal balance of spinopelvic has become a therapeutic goal of lumbosacral surgery, with increasing attention being paid by surgeons to its impact on long-term postoperative outcomes, both radiologically and symptomatic ASD [12, 13]. Natural language processing allows convenient screening of big data, and we rely on this technique to achieve intelligent medical diagnosis for medical record review and human-machine interaction from EMS [14–18]. This study focused on the risk factors for degeneration of the adjacent L4-L5 segment following MIS-TLIF of this critical lumbosacral transition region.

In our study, postoperative DA was identified as the relevant risk factor in developing both radiological and symptomatic ASD following MIS-TLIF at L5-S1. A recent clinical study published by Chung et al. suggests that the L5-S1 lordosis accounts for 40% of the entire lumbar spine and restoring alignment of this segment is an important treatment goal for spinal fusion surgery [11]. In line with previous biomechanical reports, Umehara et al. have emphasized the importance of maintaining normal lumbar lordosis after surgery for the prevention of radiological ASD, with loading and posterior shear forces from the instrumented lumbar fusion increasing vertebral stress and disc flexion-extension motion in the adjacent segment [19]. In subjects who have undergone MIS-TLIF, debridement of the interbody space, restoration of the index disc height and angle, and achievement of reliable osseointegration are goals of this procedure, so the DA, which represents the lordosis of the vertebral body, is one of the indicators used to measure the efficacy of MIS-TLIF technique. However, we prudently reviewed DA to avoid overcorrection. After 6 years of follow-up for degenerative lumbar spondylolisthesis, Liu et al. observed greater DA in the cohort with complete laminectomy and fusion, which subsequently had more frequent adjacent joint degeneration [20]. Chung and colleagues also agreed with the notion that laminectomy and DA are interactive [11]. A possible rational explanation for our conclusion that unfavorable DA correction is significantly associated with radiological ASD is that the lordosis restored by TLIF approach is usually limited compared to ALIF [21]. In other words, for patients with unstable lordosis, the implanted cage is the most direct factor that helps to correct the alignment. Although more complex anatomy has to be considered, ALIF has significant advantages by allowing a larger size of cage in this disc space. The association between DA and unfavorable postoperative functional outcomes examined in this study is consistent with coverage in [22]. The results of Kanayama et al. further discussed clinical symptoms following intervertebral fusion and noted that disc angle in flexion is an important prognostic factor [23]. Considering the predictive performance of sagittal DA in predicting complications after MIS-TLIF surgery at the L5-S1 level, including a higher DA suggesting a lower fusion rate of the operated segment [24] and a lower DA being a significant associated factor of intraoperative endplate injury [25] and fixed flat-back deformity [26], future studies need to develop individualized models of the corresponding vertebral segment when investigating the triggers of symptomatic ASD. In parallel, the pathological process of ASD requires to be defined precisely in order to preclude the interference of natural degeneration of adjacent vertebrae.

PI, the value calculated as sum of PT and SS, is an important parameter that describes the sagittal balance of spinopelvic alignment [27]. Previous studies support that a smaller PI leads to loss of upper lumbar curvature and the development of scoliosis, increased mechanical axial loading, and, in particular, adjacent disc degeneration caused by abnormal spinopelvic sequences [8, 28–30]. In our study, SS was smaller in the ASD group than in the control group, in contrast to PT, which has been shown to be a risk factor for symptomatic ASD and a marker of sagittal balance compensation, and is strongly associated with the development of radiographic ASD, but not with symptomatic ASD. Previous studies investigated multisegmental degeneration including L1 to S1; our small patient cohort was limited to single-segment MIS-TLIF at L5-S1, possibly explaining the specific association of lumbosacral fusion and PI. To further analyze the important role of spinopelvic alignment in patients with lumbar degeneration undergoing MIS-TILF, ∆PI-LL was used to evaluate adjacent segmental degeneration after imbalance in sagittal position. Following a previous study by Rothenfluh et al. PI-LL may be a comprehensive description of the lumbar and pelvic balance [8]. For patients undergoing interbody fusion, the PI-LL classification with a threshold of 10° is sufficiently credible and sensitive that the mismatched group with greater than 10° is a susceptible group for ASD, with a 10-fold higher risk of developing the disease than the matched group [8]. This interactive radiographic indicator recommends that a single surgical aim is often counterproductive: patients achieving an increase in PI should also correct LL at the same time to obtain the expected sagittal balance. This surgical consensus has been practiced by most interbody fusion procedures [28–32], but the reports after MIS-TLIF remain unclear. We found that the mismatched group of decreased PI and decreased LL was a risk factor for adjacent unfused segmental degeneration. These patients with inferior PI-LL were exposed to local biomechanical alterations and accelerated degeneration of the global lumbar spine after fusion surgery [33]. However, our study did not explain the relationship between pelvic incidence-lumbar lordosis mismatch and postoperative functional outcome. From a prospective multicenter analysis, Schwab and colleagues believe that PI-LL greater than 10° is a key factor in poor prognosis and emphasize that targeted correction of PT, sagittal vertical axis (SVA), and PI-LL are a necessary surgical purpose to improve the deformity [32]. Whether MIS-TLIF is the surgical intervention that protects patients from poor clinical outcomes even after radiographic segmental degeneration due to PI-LL mismatch still needs more investigation and validation.

The discussion described above has highlighted the adverse effects of lumbar and pelvic imbalance on adjacent segments following MIS-TLIF. However, further assessment of the predictive performance of various parameters for symptomatic ASD remains controversial. The study from Chaleat-Valayer and others indicated that chronic low back pain was more common in patients with low SS, low LL, and small PI [34]. He and colleagues reported a specific pattern of sagittal spinopelvic alignment that is associated with symptomatic ASD, including SL and PT [13]. In this study, in addition to postoperative DA, postoperative PT was another key indicator of symptomatic ASD, with worse improvement in PT in the symptomatic ASD group. To our surprise, Yamasakid and others showed that preoperative PT greater than 22.5° was a significant risk factor for a 5-fold increase in the incidence of symptomatic ASD after TILF [35], in high agreement with our study. PT has been shown to be a better predictor due to postoperative compensatory mechanisms than PI, which is virtually unchanged because of anatomical features. Vazifehdan suggests that insufficient PT correction is responsible for the unfavorable alignment pattern following single-segment posterior fusion compared to controls, with subsequent development of ASD [12]. Unfavorable PT improvement often results in restricted hip movement, increased energy expenditure, and compensatory upright posture, which exacerbates the incidence of low back pain [36, 37].

There are several weaknesses worth noting in our study. The included radiological measures are not exhaustive and there might be other contributors to adjacent segmental degeneration. However, we believe that the measurements implemented here are representative of those commonly used in practice. Second, ASD associated with MIS-TLIF was our primary concern; therefore patients were followed up for only 3 years to minimize natural degeneration. Finally, we analyzed different spinal degeneration cases at L5-S1, whose imaging findings might demonstrate different pathological changes. Although narrowing the inclusion criteria may enhance the homogeneity of the study, by presenting all surgical cases performed in the lumbosacral spine, this study reflects the real clinical practice of senior spine surgeons.

## 5. Conclusion

The results from our three-year follow-up study indicated that smaller postoperative PI, greater postoperative DA, and mismatched ∆PI-LL were significantly associated with radiological ASD. Greater postoperative PT and DA are risk factors for the unfavorable clinical symptoms in patients with radiological ASD. Surgeons can use this information to better assess which patients may require additional alignment restoration following TLIF.

## Figures and Tables

**Table 1:** Demonstrative information of the patient cohort.

	Overall	Control cohort	ASD cohort	*P* Value
No. of patients	157	141	16	
Age (years), mean ± SD	62.3 ± 7.0	62.0 ± 6.9	65.1 ± 7.7	0.098
Sex (male/female)	62/95	57/84	5/11	0.477
BMI (kg/m^2^), mean ± SD	24.4 ± 2.3	24.5 ± 2.3	23.6 ± 1.4	0.150
Smoking, *n* (%)	26 (16.6)	24 (17.0)	2 (12.5)	0.645
Diabetes mellitus, *n* (%)	29 (18.4)	26 (18.4)	3 (18.8)	0.976
Osteoporosis, *n* (%)	45 (28.7)	41 (29.1)	4 (25.0)	0.732
Diagnosis, *n* (%)				0.884
LDH	76 (48.4)	68 (48.2)	8 (50.0)	
LSS	47 (29.9)	43 (30.5)	4 (25.0)	
DS or SO	34 (21.7)	30 (21.3)	4 (25.0)	
Laminectomy, *n* (%)	109 (69.4%)	98 (69.5)	11 (68.8)	0.951
Cement augmentation, *n* (%)	17 (10.8)	14 (9.9)	3 (18.8)	0.282

*SD*: standard deviation*, BMI*: body mass index, *ASD*: adjacent segment degeneration, *LDH*: lumbar disc herniation, *LSS*: lumbar spinal stenosis*, DS*: degenerative spondylolisthesis*, OS*: isthmic spondylolisthesis.

**Table 2:** Comparison of the radiologic variables.

	Total	Control cohort	ASD cohort	*P* Value
DA (°)				
Preop	17.3 ± 4.6	17.4 ± 4.6	17.1 ± 4.4	0.829
Postop	25.1 ± 5.6	25.7 ± 5.3	19.3 ± 5.1	<0.001
LL (°)				
Preop	44.3 ± 3.2	45.7 ± 3.5	44.8 ± 2.7	0.078
Postop	53.4 ± 6.4	53.9 ± 6.2	48.5 ± 6.4	0.001
DLL (°)				
Preop	28.0 ± 5.4	27.9 ± 5.3	28.4 ± 5.7	0.720
Postop	34.2 ± 6.7	34.3 ± 6.8	33.7 ± 5.7	0.774
SS (°)				
Preop	34.3 ± 5.0	34.4 ± 5.1	34.0 ± 4.5	0.758
Postop	31.3 ± 5.1	31.4 ± 5.1	30.9 ± 5.2	0.702
PI (°)				
Preop	55.3 ± 8.6	54.8 ± 8.7	58.8 ± 7.7	0.081
Postop	53.9 ± 4.6	54.3 ± 4.6	51.6 ± 4.5	0.025
PT (°)				
Preop	21.7 ± 5.7	21.9 ± 5.7	22.3 ± 5.1	0.295
Postop	22.8 ± 5.4	22.5 ± 5.4	25.4 ± 4.6	0.044
∆PI-LL (°)				0.024
Matched group	123	114 (80.9)	9 (56.2)	
Mismatched group	34	27 (19.1)	7 (43.8)	
Cage subsidence (%)				0.625
Yes	23 (14.6)	20 (14.2)	3 (18.8)	
No	134 (85.4)	121 (85.8)	13 (81.2)	

*Preop*: preoperative, *Postop*: postoperative, *DA*: disc angle, *LL*: lumbar lordosis, *DLL*: distal lumbar lordosis, *SS*: sacral slope, *PI*: pelvic incidence, *PT*: pelvic tilt, ∆*PI-LL*: the difference value between PI and LL, *matched group*: ∆*PI-LL  < 10°, mismatched group*: ∆*PI-LL ≥ 10°*.

**Table 3:** Description of changes in DOI and VAS scores.

Oswestry disability index and the visual analogue scale	Overall	Control cohort	ASD cohort
ODI score			
Preop	64.4 (6.4–87.1)	64.5 (7.9–85.4)	63.1 (9.8–87.7)
Postop	18.3 (2–71.4)	17.7 (2.3–68.5)	24.1 (4.1–74.2)
VAS score			
Preop	6.9 (1–10)	6.9 (1–10)	6.8 (1–10)
Postop	1.9 (0–9)	1.8 (0–9)	3.5 (0–9)

**Table 4:** Univariate and multivariate logistic regression analysis of radiological ASD following MIS-TLIF.

	Univariable logistic regression analysis				Multivariable logistic regression analysis			
	OR	95% CI		*p*	OR	95% CI		*p*
		Lower	Upper			Lower	Upper	
Preop. LL	0.888	0.806	0.978	0.016	0.939	0.839	1.050	0.267
Postop. LL	0.961	0.781	0.950	0.003	0.842	0.742	0.956	0.008
Postop. DA	0.792	0.702	0.894	<0.001	0.804	0.703	0.919	0.001
Postop. PI	1.148	1.013	1.299	0.030	1.127	0.965	1.316	0.132
PI-LL								
Matched group	Ref	Ref	Ref	Ref	Ref	Ref	Ref	Ref
Mismatched group	3.284	1.123	9.604	0.030	4.370	1.015	18.816	0.048

*OR*: odds ratio, *95% CI*: 95% confidence interval.

**Table 5:** Univariate and multivariate logistic regression analysis of symptomatic ASD following MIS-TLIF.

	Univariable logistic regression analysis				Multivariable logistic regression analysis			
	OR	95% CI		*p*	OR	95% CI		*p*
		Lower	Upper			Lower	Upper	
Postop. DA	0.796	0.666	0.951	0.012	0.777	0.634	0.951	0.015
Postop. PT	1.228	1.011	1.491	0.038	1.246	1.009	1.539	0.041

*OR*: odds ratio, *95% CI*: 95% confidence interval.

## Data Availability

The dataset supporting the conclusions of this paper can be obtained from the medical electronic system in our hospital.

## List of abbreviations

ASD: Adjacent segment degeneration
MIS-TLIF: Minimally invasive transforaminal interbody fusion
PROs: Patient-reported outcomes
BMI: Body mass index
PI: Pelvic incidence
PT: Pelvic tilt
SS: Sacral slope
LL: Lumbar lordosis
DLL: Distal lumbar lordosis
SD: Standard deviation
SVA: Sagittal vertical axis
EMS: Electronic medical system
AP: Anteroposterior
CT: Computed tomography
MRI: Magnetic resonance imaging.
